# Childhood Obesity: A Multisystem Challenge Linking Hypertension, NAFLD, and Sleep Apnea

**DOI:** 10.3390/medsci14010070

**Published:** 2026-02-04

**Authors:** Martina Montagnana, Elisa Danese, Sara Bonafini, Cristiano Fava

**Affiliations:** 1 Department of Medicine—DIMED, University of Padova, 35128 Padova, Italy; 2 Laboratory Medicine, University-Hospital of Padova, 35128 Padova, Italy; 3 Clinical Biochemistry Section, Department of Engineering for Innovation Medicine, University of Verona, 37134 Verona, Italy; elisa.danese@univr.it; 4 General Medicine & Hypertension Unit, University-Hospital of Verona, 37134 Verona, Italycristiano.fava@univr.it (C.F.); 5 Department of Medicine, University of Verona, 37134 Verona, Italy

**Keywords:** cardiovascular disease, hypertension, non-alcoholic fatty liver disease, obesity, obstructive sleep apnea syndrome

## Abstract

Childhood overweight and obesity represent a major global public health emergency, with a steadily increasing prevalence over recent decades in both developed and developing countries. Approximately one fifth of children and adolescents are overweight or obese, with marked differences across ethnic groups and geographical areas. Accurate estimation of this condition is complicated by the lack of a unique and universally accepted definition of childhood obesity, which is based on different anthropometric criteria. Although body mass index (BMI) remains the most widely used tool, growing evidence indicates that abdominal obesity, assessed by waist circumference and waist-to-height ratio, is a better predictor of cardiometabolic risk, even in children with a normal BMI. Childhood obesity is associated with several comorbidities, including arterial hypertension, non-alcoholic fatty liver disease (NAFLD) and obstructive sleep apnea syndrome (OSAS). Early diagnosis and an integrated therapeutic approach are essential to reduce the risk of long-term complications. Although lifestyle modifications remain the cornerstone of treatment, new pharmacological options for pediatric obesity have been approved in recent years. This narrative review explores the impact of childhood obesity on the early development of hypertension, NAFLD, and OSAS, emphasizing the implications that can already be observed during childhood and adolescence. It examines the association between pediatric obesity and these conditions by synthesizing current epidemiological evidence, describing the underlying pathophysiological mechanisms linking excess adiposity to disease onset, and reviewing pediatric-specific diagnostic criteria as well as preventive and therapeutic strategies.

## 1. Introduction

Childhood overweight, including its severe form, obesity, is a well-known global health emergency, affecting nearly 20% of children and adolescents aged 2–19 years in the US [1].

Within ethnic groups, there are large differences in the prevalence of overweight and obesity. Accordingly, epidemiological studies performed in large United States populations demonstrated that obesity is more prevalent among American Indian and/or Native Alaskan (31.2%), non-Hispanic black (20.8%), and Hispanic (22.0%) compared with white (15.9%) and Asian children (12.8%) [2]. The pooled prevalence estimates of overweight/obesity in European preschool children during the period 2006–2016 was 17.9% (95% CI: 15.8–20.0), and the pooled prevalence estimate of obesity was 5.3% (95% CI: 4.5–6.1) [3]. The worldwide trend has particularly increased in the last few decades in both developed and developing countries [4].

An accurate estimate of the phenomenon is made difficult by the reduced standardization in the definition of obesity in childhood. While the definition of overweight and obesity in adults has well-established criteria, the definition in children is more controversial, including 110% or 120% of ideal weight for height, weight-for-height Z-scores of >1 and >2, and Body Mass Index (BMI) at the 85th, 90th, 95th, and 97th percentiles (based on international or country-specific reference populations). In most cases, childhood obesity is defined as having a BMI of ≥95th percentile for age and sex and severe obesity is defined as BMI ≥ 120% of the 95th percentile for age and sex [1]. Not only BMI but also abdominal obesity evaluated through waist circumference (WC) and waist-to-height ratio should be considered since it has been proven that, in children, abdominal obesity is a better predictor of cardiovascular risk factors than obesity evaluated using BMI [5], and that a substantial proportion of children with normal BMI have abdominal obesity [6].

Physical inactivity and an obesogenic dietary environment are the main causes of this major public health problem [7]. During the COVID-19 pandemic, some preventive measures, such as school closures and social distancing, have further aggravated the situation [8,9], making it necessary to introduce new tools, such as telemedicine, to fight “the obesity pandemic” [10].

Furthermore, obesity is also a risk factor for several related comorbidities that include pediatric obstructive sleep apnea (OSAS), dyslipidemia, hypertension, non-alcoholic fatty liver disease (NAFLD), type 2 Diabetes Mellitus (T2DM) and polycystic ovary syndrome (POS) [11]. Epidemiological studies have also demonstrated an increase in cancer risk among children and adolescents with severe obesity [12].

The timely diagnosis and management of pediatric obesity and its complications are thus of primary importance. For this reason, national and international societies have proposed screening programmes to identify children at risk of becoming future patients and intervention programmes to reduce the risk of developing chronic diseases [13]. Also, several guidelines for the evaluation and treatment of children with obesity have been published [14].

Lifestyle and behavioural modification, proposed during counselling activity, play a central role but do not seem sufficient to stop this pandemic. On the other hand, in the last few years, the Food and Drug Administration (FDA) has approved medications for children and adolescents with obesity [15].

This narrative review is aimed at highlighting the critical health problem of childhood obesity and its relationship to several related comorbidities, particularly hypertension, NAFLD and OSAS.

The literature search was conducted using the main biomedical databases, including PubMed/MEDLINE, Scopus, and Web of Science. Additional relevant articles were identified by the manual screening of reference lists from key reviews and guidelines. Search terms included combinations of keywords such as “childhood obesity”, “pediatric hypertension”, “NAFLD” and “obstructive sleep apnea”.

General inclusion criteria were as follows:

(i) Studies conducted in children and adolescents (≤18 years);
(ii) Original research articles, clinical trials, observational studies, and relevant guidelines or high-quality reviews;
(iii) Articles published in English.

Experimental animal and mechanistic studies were included only when relevant to clarify pathophysiological mechanisms, while case reports and small case series were excluded unless they provided unique mechanistic insights.

## 2. Hypertension in Obese Children

Due to the increase in the prevalence of pediatric hypertension and, as a consequence, in the risk of subclinical cardiovascular disease, in recent years there has been more attention on the increase in blood pressure (BP) in children [16].

In pediatric populations, the definition of hypertension differs significantly from that in adults. If the adult hypertension definition uses fixed cut-offs, pediatric diagnosis up to early adolescence relies on percentile charts to account for developmental changes. Accordingly, children’s BP varies with age, sex, and height. According to the 2017 American Academy of Pediatrics (AAP) Clinical Practice Guideline for Screening and Management of High Blood Pressure in Children and Adolescents, a normal BP in children aged 1–12 years is defined as systolic and diastolic BP as below the 90th percentile for age, sex, and height [17]. Elevated BP is defined between the 90th and 95th percentiles and hypertension starts at the 95th percentile or higher, as confirmed on at least three separate occasions. For adolescents aged ≥13 years, the thresholds align with adult criteria: Stage 1 hypertension is defined as ≥130/80 mmHg and Stage 2 as ≥140/90 mmHg, regardless of percentile adjustments. The European society of hypertension (ESH) guidelines also uses age-, sex-, and height-specific percentiles to define normal and elevated blood pressure in younger children, but the reference population has not excluded overweight/obese subjects as in the AAP norms [18]. Moreover, ESH applies adult fixed cut-offs later in adolescence than AAP (16 years versus 13 years) and these cut-off values are different.

The most important epidemiological results regarding the association between high BP and overweight/obesity status are presented in Table 1.

In a recently published study performed on more than 800,000 youths aged 3 to 17 years, a high-normal weight above the 60th percentile of BMI for age was also associated with an increased risk of hypertension [22]. The adjusted hazard ratio for hypertension within a maximum of 5 years was 1.26 (95% CI, 1.20–1.33) for subjects between the 60th and the 84th percentiles, compared with subjects with a baseline BMI for age in the 40th to 59th percentiles.

It is interesting to note that also in the group of children whose BMI was in the accepted normal range (a BMI in the 25th–84th percentiles), there was an increase in the risk of elevated BP as BMI increased [23].

Juonala et al. [24], by analyzing data from 6328 children of four different longitudinal studies (the Bogalusa Heart Study and the Muscatine Study conducted in the US, the Australian Childhood Determinants of Adult Health study, and the Cardiovascular Risk in Young Finns Study) observed that subjects with consistently high adiposity status from childhood to adulthood, as compared with subjects with a normal BMI, had an increased risk of hypertension (relative risk, 2.7; 95% CI, 2.2 to 3.3).

The pathophysiology of primary (essential) hypertension in overweight and obese children is complex and not yet completely understood. It includes multiple mechanisms: the secretion of hormones and cytokines, endothelial dysfunction and oxidative stress, sympathetic nervous system (SNS) activation by central mediation in the hypothalamus and local peripheral action, vascular damage, sodium and fluid retention through activation of the renin–angiotensin–aldosterone system (RAAS) [25,26].

Adipose tissue acts as an endocrine tissue that secretes hormones and cytokines with autocrine, paracrine and endocrine functions, involved in the regulation of BP [27]. Dysregulated release of adipokines, including leptin, resistin, tumour necrosis factor-α, interleukin-6, adipsin, visfatin, and adiponectin, contributes to the development of hypertension [28].

Adiponectin, a key hormone secreted by adipose tissue, plays a central role not only in glucose regulation, lipid metabolism, and insulin sensitivity, but also in vascular function by stimulating nitric oxide (NO) production in endothelial cells [29] and modulating peroxisome proliferator-activated receptor-gamma (PPARγ) activity [30]. Interestingly, PPARγ activation can suppress smooth muscle cell migration and proliferation, while promoting vasodilation. In obese children, low levels of adiponectin are associated with a higher probability of developing hypertension. Moreover, subjects with the lower plasma levels of adiponectin were those who have both hypertension and obesity [31].

However, it remains unclear whether the relationship between adiponectin and BP is only mediated by the presence of weight excess and visceral adiposity or whether adiponectin plays an obesity-independent role in BP regulation [32].

Gómez-Díaz et al. observed, in 240 Mexican children, that adiponectin concentrations were inversely associated with high BP even after adjustment for BMI and WC [33]. Accordingly, Wang et al. found, in a sample of Chinese male adolescents, that plasma adiponectin concentrations were negatively related with systolic BP values, and this association was independent of visceral adiposity [34].

As recently observed, in particular high-molecular-weight (HMW) adiponectin is associated with hypertension in obese children, being at lower circulating concentrations in obese and hypertensive subjects [35]. For this reason, it has been suggested to use HMW adiponectin levels and HMW/HOMA-IR ratio as biomarkers to identify hypertension in childhood obesity.

Among adipokines, leptin has also been identified as a link between adiposity and BP, acting as a mediator of obesity-induced hypertension [36]. Accordingly, leptin concentrations are positively associated with BP in obese children and adolescents [37]. In a cohort of 400 Japanese school-aged children, the authors demonstrated that, of the total effect of fat mass index on BP, the mediating effect of leptin accounted for 78.6% (*p* = 0.03) in boys and 42.2% (*p* = 0.11) in girls [37]. This effect of leptin on BP is thought to be mediated by SNS in blood vessels and kidneys, as supported by experimental studies showing increased epinephrine and norepinephrine secretion following leptin administration in animal models [38].

In a longitudinal study (mean follow-up: 4.5 years) including 1111 children (mean enrolment age: 10.2 years), serum leptin concentrations were statistically higher in overweight children (BMI of ≥85th percentile for age and sex) than in normal weight. Moreover, leptin and heart rate (expression of the activation of the sympathetic nervous system) showed an almost identically patterned relation to BP to that of BMI percentile and BP, thus implicating a possible mediating role for leptin [39].

In addition, in obesity, the physiological NO-mediated vasodilatory effect of leptin is impaired due to the leptin resistance [40], and thus probably contributes to the development of arterial hypertension [41].

Numerous studies observed that the release of adipokines and cytokines from adipose tissue also leads to compensatory hyperinsulinemia and, finally, to the abnormal activation of the RAAS [42]. It has been widely proven that a dysregulated RAAS has a causal role in obesity-associated hypertension [43,44]. In a retrospective study of 102 adolescents (13–16 years) with primary hypertension [45], the authors observed that patients with higher pre-treatment values of aldosterone and plasma renin activity (PRA) had a better response to angiotensin-converting enzyme inhibitor and angiotensin II receptor blocker drugs than children with lower baseline values, thus highlighting the importance of RAAS dysregulation in the development of hypertension.

Although it has not yet been proven in children, a reduction in weight in adults leads to a reduced RAAS in plasma and adipose tissue [46].

In a recently published cross-sectional Portuguese study of 313 children aged 8–9 years old, overweight/obese children (*n* = 150) demonstrated significantly higher Angiotensin-Converting Enzyme (ACE) and ACE2 activities when compared to the normal-weight subjects (*n* = 163) [47].

Autonomic dysfunction has been proposed as a key mediator in the pathogenesis of obesity-related hypertension in children, particularly through an imbalance between parasympathetic and sympathetic activity [48]. Accordingly, in obese children, an increase in heart rate and heart rate variability has been observed [49,50]. Weight status particularly influences the autonomic cardiac regulation [51]. In a study of 326 obese children aged 7 to 16 years, decreased heart rate recovery (HRR) was closely associated with prehypertension in obese children [52]. Similarly, in the study of Latorre-Román and colleagues, sedentary children with overweight and obesity showed signs of autonomic dysfunction reflected in their low cardiac vagal activity and poor chronotropic competence [51].

The increase in hypothalamus–pituitary–adrenal (HPA) axis activity observed in obese children has also been hypothesized as a link between obesity and hypertension [53]. However, salivary cortisol and cortisone levels were significantly lower in overweight or obese than in non-overweight children but no significant differences in cortisol parameters were observed between hypertensive and normotensive overweight or obese children [53]. Moreover, a recent study analyzing 38 overweight/hypertensive, 83 overweight/non-hypertensive, and 56 non-overweight/non-hypertensive children found elevated urinary cortisol and corticosterone metabolites in obese children regardless of their hypertensive status [54].

Due to the decreased absorption of magnesium from the intestine or increased excretion, overweight and obese children have lower magnesium concentrations than normal-weight children, with an inverse relationship observed between serum magnesium concentrations and BMI [55]. Low serum magnesium, by increasing intracellular calcium levels, can cause vasoconstriction and finally high BP [56].

The adipose tissue is responsible for the sequestration of fat-soluble vitamins, such as vitamin D [57]. Moreover, the adipocyte-derived hormone leptin activates a pathway that inhibits renal synthesis of the active form of vitamin D [58]. Evidence from the 2003–2006 National Health and Nutrition Examination Survey cross-sectional study showed a markedly higher prevalence of vitamin D deficiency among obese (34–56%) compared with normal-weight children (16–21%) [59]. In obese children, an interesting association was observed between low levels of vitamin D and a higher BP burden [60], especially at night [61]. Vitamin D is known to modulate the renin–angiotensin system [62] and to limit the proliferation and migration of vascular smooth muscle cells under endothelial stress [63]. Moreover, the treatment with vitamin D supplementation in vitamin D-deficient overweight and obese children has proven effective in reducing BP [64].

## 3. NAFLD in Obese Children

The prevalence of NAFLD, which represents the leading cause of chronic liver disease in children, is estimated to be approximately between 3% and 10% in the general pediatric population but it exceeds 40% in obese children [65]. Reported prevalence rates vary widely according to the characteristics of the population studied and the accuracy of the diagnostic test [65,66]. 

In 2017, specific recommendations regarding the diagnosis and treatment of NAFLD in children were published [67]. Even if a liver biopsy represents the gold standard for determining the presence and severity of NAFLD, in a screening strategy, clinical practice guidelines recommend measuring ALT concentration from the age of 9–11 in children with BMI ≥ 85th percentile and additional risk factors, such as central obesity, insulin resistance, prediabetes and diabetes, dyslipidemia, sleep apnea or family history of NAFLD [67]. According to NASPGHAN guidelines, sex-specific upper limits of normal for ALT should be used, with values of >22 U/L in girls and >26 U/L in boys considered abnormal, and persistent ALT elevation for more than 3 months, particularly >2 times the upper limit of normal, warranting further evaluation for NAFLD [67].

In several studies, a strong positive relationship between fatty liver prevalence and obesity was demonstrated [68,69,70]. NAFLD is more frequent in males than females [71,72,73], probably due to the antioxidant effect of estrogen, which limits fatty acid accumulation in hepatocytes in females [72]. Moreover, population-based studies also observed that prevalence is related to race/ethnicity, with NAFLD being more common in Hispanic and Asian than in White or Black children [74].

NAFLD in children should be suspected in overweight or obese subjects with persistent elevation of serum alanine aminotransferase (ALT) after the exclusion of other causes of liver disease [75,76].

Even if the sensitivity of ALT in detecting NAFLD or non-alcoholic steatohepatitis (NASH) is lower than 60%, as reported in the study of Schwimmer and colleagues [77], it is the most cost-effective non-invasive screening test for obese children. However, it is recommended to use different cut-offs according to the age of the child and according to the test kit used for measurement [78,79]. Importantly, pediatric NAFLD is a diagnosis of exclusion, requiring systematic evaluation to rule out secondary causes of elevated transaminases and hepatic steatosis, including viral hepatitis, autoimmune liver disease, Wilson disease, α1-antitrypsin deficiency, medication-induced liver injury, and significant alcohol consumption in adolescents [67]. Liver biopsy remains the reference standard for diagnosing nonalcoholic steatohepatitis and assessing fibrosis but is reserved for selected children with high risk of advanced disease or diagnostic uncertainty due to its invasive nature [67].

The pathogenesis of the disease is complex, multifactorial, and not yet completely understood; it involves several organs and tissues. According to the “two hits hypothesis”, the first hit is represented by hyperinsulinemia and insulin resistance induced by a hypercaloric diet and a sedentary lifestyle in genetically predisposed children; the “second hit” includes the release of inflammatory cytokines and adipokines, together with mitochondrial dysfunction and oxidative stress [80]. The consequence of the first hit is hepatic triglyceride accumulation (hepatic steatosis), while pathogenetic factors involved in the second hit drive the progression of the disease with inflammation, fibrosis, and finally, cirrhosis. More recent evidence supports the “multiple-hit model”, in which obesity and insulin resistance, by inducing liver fat accumulation, are still the first and necessary actors in the pathogenesis of NAFLD [81,82]. However, the interaction of genetic and environmental factors (for example, a diet rich in fructose, mostly metabolized in the liver) [83] successively leads to the release of proinflammatory cytokines, both systemically and locally, in areas the liver other, leading to gut–liver axis (GLA) dysfunction [84]. According to the multiple-hit model, other organs, such as the pancreas, can also be involved in NAFLD development and progression [85]. It is interesting to highlight that in overweight/obese children with NAFLD, pancreatic fat is increased compared with diseases without liver involvement [86], and it shows an independent positive association with BMI [87].

As occurs in adulthood, in children, there are also different degrees of the pathology, ranging from isolated hepatic steatosis to an advanced form of NASH with inflammation and fibrosis, which may lead to cirrhosis and end-stage liver disease [88].

Regarding genetic predisposition, several polymorphisms have been studied. However, among variants that genetically predispose to NAFLD, the apparently most interesting are polymorphisms in the patatin-like phospholipase containing domain 3 (PNPLA3), in the G-protein-coupled-receptor 120 (GPR120) and in the transmembrane 6 superfamily member 2 (TM6SF2) genes [89]. PNPLA3 encodes for the enzyme adiponutrin, which shows lipolytic activity in the triglycerides in both liver and adipose tissue [90].

A specific polymorphism of this gene, the rs738409[G], encoding an isoleucine-to-methionine substitution at the amino acid position 148 (I148M), confers increased susceptibility to hepatic steatosis, not only in adults [91] but also early in life, in obese children and adolescents [92,93]. Moreover, morphological analysis has demonstrated that this polymorphism is associated with smaller adipocytes in the subcutaneous adipose tissue and, consequently, with the overflow of free fatty acids to the liver, in which they accumulate as triglycerides [92]. Accordingly, in an Italian study that involved 1048 obese children [94], the authors found statistically significant higher ALT and aspartate transaminase (AST) levels and a higher extent of abdominal fat in children carrying the 148M allele.

The *GPR120* gene encodes for a receptor of unsaturated long-chain free fatty acids and plays a key role in sensing dietary fat [95]. It has been reported that the *GPR120* R270H variant is associated with lower expression of the GPR120 receptor on Kuppfer cells [96]. Accordingly, by enhancing inflammation in adipose and hepatic tissues, the variant is associated with higher ALT concentrations in children and adolescents with obesity in comparison to normal-weight subjects [95].

Finally, the rs5852926 variant of the *TM6SF2* gene, which results in the substitution of glutamate by lysine at residue 167, is associated with a markedly decreased TM6SF2 protein expression in the liver of NAFLD patients, and consequently with reduced very-low-density lipoprotein (VLDL) secretion, finally leading to increased liver fat accumulation [97].

Interestingly, by enrolling 152 obese children and adolescents with biopsy-proven NAFLD and increased liver enzymes, Nobili et al. [98] tested a 4-polymorphism risk score to predict the risk of NAFLD. The score included the above-mentioned PNPLA3 rs738409, the rs4880 C>T SOD2, which encodes for the mitochondrial enzyme manganese-dependent superoxide dismutase, the rs3750861 G>A of the Kruppel-like factor 6 (KLF6) gene, and lipin 1 (LPIN1) rs13412852 C>T polymorphisms [99]. This genetic risk score showed better performance (area under the receiver-operating characteristic curve [AUC] 0.75; 95% confidence interval [CI] 0.67–0.82, *p* < 0.0001) in predicting NASH than a clinical risk score based on age, AST, and diastolic blood pressure (AUC 0.66, 95% CI 0.57–0.75).

More recently, several studies have shown that gut microbiota dysregulation (dysbiosis) can contribute to the pathogenesis of NAFLD [100,101,102] and that the intestinal microbiome has an important role in both the development and progression of NAFLD, by altering gut–liver homeostasis and disrupting the gut barrier [103]. By analyzing microbiome composition in fecal samples of 25 children with NAFLD, 25 with NASH, and 25 obese children without NAFLD, Pan and colleagues observed that anti-inflammatory and probiotic contents (e.g., Faecalibacterium, Akkermansia, and Bifidobacterium_adolescentis) were significantly decreased in NAFLD, and that butyrate-producing bacteria, such as Clostridia and Bacteroidia, were significantly decreased in NASH, suggesting a role of different species in the progression of hepatic disease [103].

Finally, hypertension is also implicated in the evolution of NAFLD in obese children [104]. Accordingly, a high BP may lead to altered intrahepatic splanchnic circulation and increased intrahepatic vascular resistance, which could cause NAFLD to develop into NASH, cirrhosis and portal hypertension [105].

## 4. OSAS in Obese Children

OSAS is a chronic disease characterized by recurrent episodes of partial or complete upper-airway collapse and obstruction during sleep. These episodes are accompanied by intermittent drops in oxygen levels, interruptions in sleep patterns, and symptoms such as disruptive snoring and daytime sleepiness [106].

The prevalence of OSAS in the pediatric age ranges from 1 to 59% in different populations [107,108], depending on age, gender, anatomical factors, and underlying clinical conditions. The role and impact of other risk factors, such as parental smoking [109] and asthma [110], are rather controversial and need further investigations to be proven.

Obesity has been widely shown to be one of the most important risk factors for OSAS in children [111]. Accordingly, the severity of the respiratory disease correlates with weight [112] and particularly with visceral adiposity [113]. More recently, a large study conducted in China involving over 5000 children and adolescents younger than 18 years found that more than 10% of participants were at high risk for OSAS according to the Pediatric Sleep Questionnaire. Excess body weight, particularly abdominal obesity, was associated with an increased risk of OSAS [114].

The main characteristics of studies investigating obesity and OSAS in children are reported in Table 2.

Excess weight can contribute to the development and worsening of OSAS due to the accumulation of fat, particularly around the neck and throat area [123] but also around the abdomen, thus influencing diaphragmatic function and decreasing lung volume and traction on the pharynx during sleep [124]. Accordingly, by assessing the abdominal visceral and subcutaneous adipose tissue areas in 19 obese children using magnetic resonance imaging (MRI), Canapari et al. found that only the visceral fat area was strongly predictive of mean apnea–-hypopnea index (AHI) (r(2) = 0.556; *p* = 0.003), thus correlating with the severity of OSAS [115]. Obesity and increased neck and WC are associated with poorer sleep quality in pediatric OSAS, even after adenotonsillectomy [116].

Even if polysomnography (PSG) or a sleep study is the gold standard for a specific diagnosis of OSAS [125], it is not simple to perform it in children’s populations. Therefore, different approaches have been proposed, such as questionnaires, pulse oximetry, and nap PSG, obtaining different prevalence estimates [126]. Moreover, even when applying PSG, heterogeneous results are obtained. Alonso-Álvarez and colleagues, investigating 248 Spanish children (3–14 years) with a BMI in the ≥95th percentile for the age and sex of the NANOS cross-sectional, prospective, multicenter study, observed that the prevalence of OSAS ranged from 21.5% to 39.5% depending on the different respiratory index used to investigate the disease [117]. A meta-analysis restricted to polysomnography-based studies confirms obesity and tonsillar hypertrophy as major risk factors for OSAS [118].

Arens and colleagues [119], by using MRI to study the upper airway and abdomen of 22 obese children with OSAS and 22 matched obese children without OSAS, observed that children affected by the disease had larger upper airway lymphoid tissues, larger the parapharyngeal fat pads, and increased abdominal visceral fat but not subcutaneous fat.

Adenoid hypertrophy and tonsillar hypertrophy, evidenced by nasal pharyngoscopy, have been demonstrated to be important risk factors for OSAS, by causing significant airway obstruction [127] not only in obese but also in non-obese children [128]. As proof of this, in a recently published study on 1550 children who underwent PSG, of whom 852 were affected by OSAS, the authors reported that in the multivariate logistic regression analysis, adenoid hypertrophy (OR:1.835, 95% CI: 1.482–2.271) and tonsil hypertrophy (OR: 1.283, 95% CI: 1.014–1.622), but not obesity, were independently associated with the risk of pediatric OSAS (*p* < 0.05) [120].

It is thus hypothesized that different mechanisms can induce the proliferation of lymphadenoid tissues and that there could be a bidirectional influence between lymphatic tissue and fat tissue [129]. However, evidence from systematic reviews, RCTs, and large cohort studies on pediatric OSAS highlights persistent OSAS after adenotonsillectomy (especially in obese children) and emerging non-surgical treatments (e.g., orthodontic interventions, myofunctional therapy). Thus, there is a need for individualized, multidisciplinary care in obese children with OSAS [130].

Obesity is a state of chronic low-grade inflammation in which adipokines are released by adipose tissue, which can affect the tone and function of the upper-airway muscles, making them more prone to collapse during sleep [131]. Moreover, the increased expression of pro-inflammatory mediators, such as leukotrienes and prostaglandins, could promote the proliferation of adenotonsillar tissues [132].

Lower respiratory tract infections are associated with OSAS and may mechanically contribute to the development of pediatric OSAS [133,134]. These viruses can also remain and affect lymphoid tissue by inducing lymphoproliferative and inflammatory responses in the nasopharynx, playing a role in perpetuating lymphoid proliferation over time [134].

Leptin concentrations are increased in children with OSAS, being more elevated in obese than in non-obese children, and they correlate with the severity of the disease [135]. The study of Tauman and colleagues [121], performed on 130 children, demonstrated that serum leptin levels were significantly increased in children with OSAS independently of obesity, thus suggesting that hypoxemia of OSAS could itself further augment leptin release from adipose tissue [136].

Since not all obese children will develop OSAS, it has been hypothesized that other factors, such as genetic predisposition, can also play a role. OSAS is more frequent in African Americans than in Caucasian subjects. Accordingly, in the Jackson Heart Sleep Study enrolling 852 African Americans participants, a high prevalence of objectively measured OSAS was observed among this group [137]. Anatomic features like neck circumference and the increased soft tissue in the upper airway could play a role in explaining the differences among races [137]. A genetic predisposition to OSAS is also evidenced by the fact that there is a certain familiarity in the development of the disease [138]. Obese children at risk for OSAS have a high probability of having parents affected by OSAS [122]. In a recently published study, Campbell et al. by enrolling 25 children with a parent diagnosed with OSA (P-OSA) and 29 age and gender-matched children with parents at low risk for OSA (P-NOSA), found that 44% of P-OSA but none P-NOSA children were overweight or obese [122]. Many obese children have sedentary behaviour and poor diet, and both these habits can contribute to overall poor tone in the muscles of the upper airway [139] that can contribute to the development of OSAS in these children [140].

## 5. Obesity and Cardiovascular Damage

Cardiovascular risk factors during adolescence, including obesity, are associated with an increased cardiovascular risk in adulthood and early vascular damage detectable by non-invasive techniques. A study by Urbina’s group found that the sum of cardiovascular risk factors is associated with left ventricular mass, pulse wave velocity, and renal function, decreased peak longitudinal strain, urine albumin-to-creatinine ratio, and echocardiographic parameters of diastolic dysfunction [141].

In a sample of obese and overweight children and adolescents, hemodynamic parameters were strictly correlated with indices of early vascular damage. Additionally, both BMI and WC were associated with carotid distensibility and stiffness index, as measured by digital photoplethysmography [142].

Genovesi and colleagues found that higher left ventricular mass (LVM) is especially associated with measures of obesity. However, lifestyle modifications can improve early cardiac damage [143,144].

## 6. Non-Pharmacological Management Strategies

Even if there is not clear evidence of long-lasting efficacy [145], non-pharmacological management strategies for the treatment of obesity should be pursued in all children and adolescents with overweight and obesity [14,146]. Lifestyle modifications, such as restricting caloric intake, improving the quality of food, and increasing physical activity, are the mainstays of therapy. Supportive counselling is fundamental to achieving results and should be provided at the highest level of intensity acceptable to the patient and their family and feasible in the available clinical setting. Engaging both the children and their families is important to promote a healthy eating environment, a balanced diet, and a physical activity programme. For higher grades of obesity (i.e., BMI > 120% of the 95th percentile or BMI ≥ 35, whichever is lower), when non-pharmacological strategies do not yield results, pharmacological and surgical treatments should be considered [14,146].

Several low-quality trials, including those featured in systematic reviews and meta-analyses, have explored interventions involving diet, physical activity, or behaviour in pre-school and primary school children. However, the results are controversial, with only some studies showing a benefit in reducing z-BMI [147,148,149]. These studies are highly heterogeneous in several respects, including the type of dietary intervention used. Some studies implemented hypocaloric, balanced diets, typically aiming for modest energy reduction rather than strict calorie targets. Others adopted low-fat dietary approaches, focusing on reducing total fat and energy-dense foods, particularly in older trials. In contrast, several interventions relied on healthy eating or dietary education models, emphasizing the reduction in sugar-sweetened beverages and unhealthy snacks, increased intake of fruits, vegetables, and whole foods, and improved meal structure. Mediterranean-style dietary patterns were only occasionally referenced and were rarely implemented as clearly defined or closely monitored interventions. Moreover, dietary strategies were usually delivered as part of multicomponent interventions combined with physical activity and behavioural approaches, rather than as standalone dietary prescriptions [147,148,149].

A more recent meta-analysis, focusing on measures of central obesity such as WC, waist-to-height ratio, waist-to-hip ratio, and WC z score, in children and adolescents aged 5–18 years old, included 8183 participants. The authors found that multicomponent lifestyle and behavioural interventions, encompassing dietary education to reduce unhealthy diet and promote healthy food intake, daily physical activity, and limit screen time significantly reduced WC; single-component approaches showed no significant effect [150].

Family-based behavioural treatments that implement a variety of behavioural techniques to develop healthy eating, physical activity, and parenting behaviours within families can led to an improvement in weight, even in children not directly treated [151].

Finally, eHealth and mobile health (mHealth) interventions using digital tools, i.e., smartphones, apps, and videogames, to promote behaviour change programmes showed encouraging results regarding weight loss through an improvement in diet and physical activity [152].

Sufficient data only exist for adults to compare the specific effects of 14 popular named dietary programmes that vary in macronutrient composition, as demonstrated in the network meta-analysis by Ge and colleagues [153]. This systematic review included 121 trials encompassing 21,942 patients. It found that while weight reduction following some dietary patterns is significant, the effect size is modest and durability beyond 12 months is limited. However, the more consistent reductions in blood pressure values and (low-density lipoprotein) LDL-cholesterol achieved with some diets, including the Mediterranean diet, could be of clinical importance.

Educational interventions aimed at parents and caregivers play a crucial role, as early-life habits strongly influence long-term weight trajectories. In addition, school-based programmes and public health policies are essential to create supportive environments that facilitate healthy choices for children.

## 7. Pharmacological Management Strategies

This review does not aim to provide a detailed presentation of pharmacological strategies for obese children and adolescents. For comprehensive information on this topic, other well-conducted reviews or guidelines should be consulted [14,15,146,154]. Drugs such as phentermine (withdrawn in Europe by the European Medicines Agency [EMA]), metformin, and orlistat are available but have not produced satisfactory results for stable weight reduction. Glucagon-like peptide 1 (GLP-1) analogues, which primarily act on satiety, have revolutionized the field. They have consistently demonstrated significant weight reduction in a considerable portion of obese adults when used long-term. Recently, their use has extended to adolescents without type 2 diabetes, yielding promising results.

Liraglutide at a dose of 3.0 mg subcutaneously once daily to obese adolescents (ages 12–17) alongside lifestyle therapy, in a 56-week randomized, double-blind, placebo-controlled trial, demonstrated slight superiority over placebo in terms of changes from baseline in BMI standard-deviation scores. Additionally, twice as many adolescents in the liraglutide group achieved at least a 5% reduction in BMI compared to the placebo group (43.3% vs. 18.7%) [155].

In a more recent randomized controlled trial (RCT) involving adolescents with obesity or overweight along with at least one weight-related comorbid condition, participants received either a once-weekly 2.4 mg dose of subcutaneous semaglutide or a placebo for 68 weeks. The mean reduction in BMI from baseline was significantly greater in the semaglutide group. Additionally, 73% of participants in the semaglutide group achieved at least a 5% reduction in BMI, compared to only 18% in the placebo group [156]. In a recent meta-analysis including 574 participants, the effects of GLP-1 analogues in children and adolescents are limited, with an average decrease in BMI of −1.24 kg/m^2^ and z-BMI of −0.14 [157]. However, these agents show advantages over previous pharmacological treatments, encompassing both efficacy and a lower frequency of adverse effects. The most frequent adverse effect was nausea, with a doubled risk compared to other treatments.

A meta-analysis summarized the findings about RCTs using GLP-1 receptor agonists for the treatment of pediatric obesity, excluding diabetes. After the inclusion of eight studies with a total of 715 patients, semaglutide, exenatide or liraglutide brought to a significant decrease in weight-related measures and systolic blood pressure but not in lipid and glucose parameters. The incidence of gastrointestinal side effects was high [158]. In general, the effect size on weight, WC and BMI is clinically significant albeit not extremely large, and in the meta-analysis the effect size was −5.54 kg, −4.30 cm, and −2.62 kg/m^2^, respectively, with no real difference between molecules. On the other hand, GLP-1 receptor agonists were associated with higher rates of nausea (42.1% vs. 16.4%) and vomiting (37.9% vs. 6.8%) compared with placebo, while diarrhea (23.6% vs. 17.1%) and upper abdominal pain (11.0% vs. 12.8%) did not differ significantly, with high-certainty evidence and no heterogeneity [159]. Rarer but more serious adverse effects, such as gallbladder disease (e.g., cholelithiasis/cholecystitis) and acute pancreatitis, were signalled in adults but not in children and adolescents [159].

Both the EMA and the FDA have approved at least one GLP-1 receptor agonist for use in adolescents aged 12 years and older with obesity, to be prescribed as an adjunct to a reduced-calorie diet and increased physical activity [160,161].

In conclusion, obesity in children and adolescents provokes deleterious metabolic and hemodynamic consequences and can cause hypertension, NAFLD, and/or OSAS even during childhood. If left untreated, obesity directly contributes to the development and progression of these complications and related pathologies. Consequently, the cardiovascular system is challenged early in life, showing early signs of impairment.

## Figures and Tables

**Table 1:** Summary of epidemiological studies examining the risk of high blood pressure/hypertension in overweight/obesity children and adolescents.

Ref.	Study	No. of Subjects	Study Design	Age Range, Years	BMI/Weight Status	Main Findings
[19]	Kit, 2015	1616	Cross-sectional	8–17	Normal weight: BMI 5th to <85th percentile (*n* = 1002); Overweight: BMI 85th to <95th percentile (*n* = 267); Obese: BMI ≥ 95th percentile (*n* = 347).	Prevalence of either high or borderline high BP was 8.4% [5.9–11.5] in normal weight vs. 12.8% [8.6–18.1] in overweight vs. 18% [12.0–25.4] in obese children.
[20]	Rutigliano, 2021	489	Cross-sectional	5–17	Overweight: BMI ≥ 85th and <95th percentile; Obese: BMI ≥ 95th percentile.	Children with elevated blood pressure increased from 12.5% with the 2004 AAP to 23.1% with the 2017 AAP criteria (*p* < 0.001). AAP guidelines identify more children with hypertension compared with previous definitions, especially in obesity.
[21]	Parker, 2016	101,606	Longitudinal	3–17	Low healthy: BMI 5th–49th percentile (*n* = 30,482); High healthy: BMI 50th–84th percentile (*n* = 38,610); Overweight: BMI 85th–94th percentile (*n* = 16,257); Obese: BMI 95th–98th percentile (*n* = 12,193); Severely obese: BMI ≥ 99th percentile (*n* = 4064).	Children 3–11 years: mean BP percentile associated with BMI was 41.9, 45.7, 50.6, 57.5 and 63.8 in low healthy, high healthy, overweight, obese and severely obese categories. Adolescents 12–17 years: mean BP percentile associated with BMI was 51.6, 52.6, 54.8, 58.1 and 63.5 in low healthy, high healthy, overweight, obese and severely obese categories.
[22]	Koebnick, 2023	801,019	Retrospective	3–17	Underweight: BMI < 5th percentile (*n* = 25,880); Low healthy weight: BMI ≥ 5th to <40th percentiles (*n* = 166,859); Medium healthy weight: BMI ≥ 40th to <60th percentiles (*n* = 128,176); High healthy weight: BMI ≥ 60th to <85th percentiles (*n* = 215,993); Overweight: BMI ≥ 85th to <95th percentiles (*n* = 132,464); Moderately obese: BMI ≥ 95th to <97th percentiles (*n* = 41,465); Severely obese: BMI ≥ 97th percentile (*n* = 90,182).	Adjusted HRs [95% CI] for incidence of hypertension were: 1.26 [1.20–1.33], 1.91 [1.81–2.00], 2.77 [2.61–2.94], 4.94 [4.72–5.18] for high healthy weight, overweight, obese and severely obese categories.
[23]	Wang, 2020	58,899	Cross-sectional	6–18	<5th (*n* = 4551), 5th–24th (*n* = 10,824), 25th–49th (*n* = 11,445), 50th–74th (*n* = 11,372), 75th–84th (*n* = 5066), 85th–94th (*n* = 6561), 95th–98th (*n* = 5202), ≥99th (*n* = 3878) percentile subgroups according to the sex- and age-specific percentiles.	The prevalence of elevated BP increased from 7.9% in <5th percentile subgroup to 16.2% in ≥99th percentile subgroup, and the prevalence of high BP increased from 6.0% in <5th percentile subgroup to 19.2% in ≥99th percentile subgroup. Compared with the 5th–24th percentile subgroup, the ORs for high BP were 1.27 (95% CI, 1.14–1.41) in the 5th–49th percentile subgroup, 1.55 (95% CI, 1.39–1.73) in the 50th–74th percentile subgroup, and 2.17 (95% CI, 1.92–2.46) in the 75th–84th percentile subgroup, respectively. The ORs for elevated BP were 1.21 (95% CI, 1.10–1.32), 1.55 (95% CI, 1.42–1.69), and 1.80 (95% CI, 1.62–2.01), respectively.
[24]	Juonala, 2011	6328	Longitudinal	3–18	Group I (*n* = 4742) subjects with a normal BMI in childhood who were nonobese as adults; Group II (*n* = 274) subjects overweight or obese in childhood but nonobese as adults; Group III (*n* = 500) subjects overweight or obese in childhood and obese as adults; Group IV (*n* = 812) subjects with a normal BMI in childhood who were obese as adults.	The RRs for hypertension in II, III and IV groups were 0.9 (95% CI, 0.6–1.4), 2.7 (95% CI, 2.2–3.3), 2.1 (95% CI, 1.7–2.4), respectively.

AAP, American Academy of Pediatrics; BMI, body mass index; BP, blood pressure; CI, confidence interval; HR, hazard ratio; OR, odd ratio; RR, relative risk. Obese and overweight children have a higher prevalence of elevated BP than normal-weight children [19,20], being reported from 4 to 14% in overweight and from 11 to 23% in obese children [20]. Accordingly, in a US cross-sectional retrospective study, odds ratios for hypertension were 4 and 2 in children with severe obesity and mild obesity compared to non-overweight children [21].

**Table 2:** Main characteristics of studies investigating obesity and obstructive sleep apnoea syndrome (OSAS) in children.

Ref.	Study	Population	Age, Years	Weight-Related Parameters	Main Findings
[114]	Liao, 2024	5000 school-aged children: Males: 49.5% at low risk for OSAS, 60.0% at high risk for OSAS Females: 50.5% with low risk for OSAS; 40.0% at high-risk for OSAS	11.0 ± 3.0 children with low risk for OSAS; 11.1 ± 2.9 children with high-risk for OSAS	Overweight: 19.7% children at low risk for OSAS; 22.9% children at high risk for OSAS. Obesity: 21.6% children at low risk for OSAS; 33.5% children at high risk for OSAS	11.4% children were at high risk for OSAS, assessed by Pediatric Sleep Questionnaire. Overweight, obesity, and abdominal obesity significantly increased OSAS risk.
[115]	Canapari, 2011	Obese children (BMI > 95th percentile), *n* = 31 (14 M, 17 F).	12.6 ± 3.0	39.5 ± 11.2 kg/m^2^; 35.4 ± 5.8 kg/m^2^ in non-OSAS; 43.9 ± 13.9 kg/m^2^ in OSAS patients.	Forty-eight percent patients had OSAS at polysomnography (mean AHI: 6.26 ± 6.77 events/h). Visceral fat area, measured by magnetic resonance imaging, was strongly predictive of AHI.
[116]	Ramírez-Contreras, 2025	407 children with OSAS, (51.4% F, 48.6% M).	6.5 ± 1.4	Obesity: 33.2%.	Children with obesity had lower sleep efficiency and higher sleep latency compared with those without obesity. Higher neck circumference was associated with lower total sleep duration.
[117]	Alonso-Álvarez, 2014	Obese children (BMI > 95th percentile), *n* = 248 (113 F, 135 M).	10.8 ± 2.6 (range 3–14)	BMI 28.0 ± 4.7 kg/m^2^; BMI percentile 96.8 ± 0.6 (95–98).	Prevalence of OSAS ranged from 21.5% to 39.5%. In 48% children, no adenoid hyperplasia, and in 57% no tonsillar hyperplasia was found.
[118]	Saporiti, 2025	3482 children (meta-analysis from nine observational studies).	1–19	Obese children *n* = 2752 (79%).	Obesity and tonsillar hypertrophy are associated with OSAS (diagnosed by polysomnography) (RR: 1.42; 95% CI: 1.20–1.68; RR: 1.61; 95% CI: 1.35–1.92, respectively).
[119]	Arens, 2010	Obese children (BMI > 95th percentile); n = 22 with OSAS (14 M, 8 F), *n* = 22 without OSAS (14 M, 8 F).	12.5 ± 2.8 OSAS; 12.3 ± 2.9 controls	34.6 ± 8.3 kg/m^2^ (2.4 ± 0.4 Z-score) OSAS; 32.4 ± 6.9 kg/m^2^ (2.3 ± 0.3 z-score) controls.	Upper-airway lymphoid hypertrophy is significantly greater in obese children with OSAS and does not correlate with obesity.
[120]	Xiao, 2022	1550 children (852 with OSAS at polysomnography and 698 with primary snoring). F 628 (40.5%). M 922 (59.5%).	5.0 (3.9–6.4) in OSAS children; 5.1 (4.1–6.6) in primary snoring children.	Obese children *n* = 128 (12%).	Adenoid hypertrophy (OR:1.835, 95% CI: 1.482–2.271) and tonsil hypertrophy (OR: 1.283, 95% CI: 1.014–1.622), but not obesity, were independently associated with the risk of pediatric OSAS.
[121]	Tauman, 2007	130 children.	8.2 +/− 2.8	Obese children *n* = 51 (39%).	Leptin concentration is higher in obese children and in children with sleep disordered breathing: possible effect of hypoxemia on leptin levels
[122]	Campbell, 2021	25 children with parents with OSA (P-OSA) vs. 29 children with parents at low-risk for OSA (P-NOSA) F 56%, M 44% in P-OSA; F 55.4% M 44.8% in P-NOSA,	7.9 ± 2.8 in P-OSA; 8.4 ± 3.0 in P-NOSA	BMI: 19.5 ± 5.5 kg/m^2^ P-OSA; BMI 16.95 ± 2.1 kg/m^2^ P-NOSA.	BMI was higher in the P-OSA group (19.5 ± 5.7 vs. 16.95 ± 2.08 kg/m^2^); 44% of the P-OSA group were overweight or obese, while none of the P-NOSA group were overweight or obese.

BMI, body mass index; OSAS, obstructive sleep apnoea syndrome; AHI, apnoea hypopnea index.

## Data Availability

No new data were created or analyzed in this study.

## Abbreviations

The following abbreviations are used in this manuscript:

AAP	American Academy of Pediatrics
ACE	Angiotensin-Converting Enzyme
ALT	Alanine Aminotransferase
AHI	Apnea–Hypopnea Index
AST	Aspartate Transaminase
AUC	Area Under the Receiver-operating Characteristic curve
BMI	Body Mass index
BP	Blood Pressure
CI	Confidence Interval
EMA	European Medicines Agency
ESH	European Society of Hypertension
FDA	Food and Drug Administration
GLA	Gut–Liver Axis
GLP-1	Glucagon-like peptide 1
GPR120	G-protein-coupled-receptor 120
HMW	High Molecular Weight
HOMA	Homeostatic Model Assessment
HPA	Hypothalamus–Pituitary–Adrenal
HRR	Heart Rate Recovery
KLF6	Kruppel-like factor 6
LDL	Low-Density Lipoprotein
LPIN1	Lipin 1
LVM	Left Ventricular Mass
MRI	Magnetic Resonance Imaging
NAFLD	Non-Alcoholic Fatty Liver Disease
NASH	Non-Alcoholic Steatohepatitis
NO	Nitric Oxide
OSAS	Obstructive Sleep Apnea Syndrome
PNPLA3	Patatin-Like Phospholipase containing domain 3
POS	Polycystic Ovary Syndrome
PPARγ	Peroxisome Proliferator-Activated Receptor-gamma
PRA	Plasma Renin A ctivity
PSG	Polysomnography
RAAS	Renin–Angiotensin–Aldosterone System
RCT	Randomized Controlled Trial
SNS	Sympathetic Nervous System
T2DM	Type 2 Diabetes Mellitus
TM6SF2	Transmembrane 6 Superfamily Member 2
VLDL	Very-Low-Density Lipoprotein
WC	Waist circumference
